# Depth-dependent functional MRI responses to chromatic and achromatic stimuli throughout V1 and V2

**DOI:** 10.1016/j.neuroimage.2020.117520

**Published:** 2020-11-01

**Authors:** Karen T. Navarro, Marisa J. Sanchez, Stephen A. Engel, Cheryl A. Olman, Kimberly B. Weldon

**Affiliations:** a Department of Psychology, University of Minnesota, 75 E River Rd, Minneapolis, MN 55455, United States; b Department of Psychiatry and Behavioral Sciences, University of Minnesota, 2450 Riverside Ave f275, Minneapolis, MN 55454, United States; c Center for Magnetic Resonance Research, University of Minnesota, 2021 6th St SE, Minneapolis, MN 55455, United States

**Keywords:** Cortical mapping, Cortical layers, High-resolution imaging, Magnocellular and parvocellular pathway, Depth-dependent fMRI

## Abstract

In the primate visual system, form (shape, location) and color information are processed in separate but interacting pathways. Recent access to high-resolution neuroimaging has facilitated the exploration of the structure of these pathways at the mesoscopic level in the human visual cortex. We used 7T fMRI to observe selective activation of the primary visual cortex to chromatic versus achromatic stimuli in five participants across two scanning sessions. Achromatic checkerboards with low spatial frequency and high temporal frequency targeted the color-insensitive magnocellular pathway. Chromatic checkerboards with higher spatial frequency and low temporal frequency targeted the color-selective parvocellular pathway. This work resulted in three main findings. First, responses driven by chromatic stimuli had a laminar profile biased towards superficial layers of V1, as compared to responses driven by achromatic stimuli. Second, we found stronger preference for chromatic stimuli in parafoveal V1 compared with peripheral V1. Finally, we found alternating, stimulus-selective bands stemming from the V1 border into V2 and V3. Similar alternating patterns have been previously found in both NHP and human extrastriate cortex. Together, our findings confirm the utility of fMRI for revealing details of mesoscopic neural architecture in human cortex.

## 1. Introduction

The visual system uses parallel processing to transmit visual input from the retina to the visual cortex (Desimone et al., 1985; Maunsell, 1987; Mishkin et al., 1983; Shipp and Zeki, 1985; Yabuta et al., 2001). Past research has demonstrated that at least three major pathways work together to transmit this information: the parvocellular (P), magnocellular (M) and koniocellular (K) pathways (Livingstone and Hubel, 1988; Norton and Casagrande, 1982; Schiller and Logothetis, 1990; Silveira et al., 2004; for review, Callaway, 2005). All three pathways begin with specific subtypes of ganglion cells that project from the retina to the lateral geniculate nucleus (LGN) and continue from the LGN to the primary visual cortex (V1) (Dacey and Lee, 1994; for review, Kandel et al., 2000; Stone, 2013). The neuronal sensitivity profiles in each pathway overlap, so no visual experience will drive a single pathway in isolation. In the present experiment, color and luminance contrast and temporal frequency were manipulated to create stimuli that would minimize responses in the K pathway and differentiate between responses in putative P and M pathways in early visual areas.

The primary goal of the present work was to use depth-dependent fMRI (e.g. Huber et al., 2017; Kok et al., 2016; Olman et al., 2012; Olman et al., 2018 Uğurbil et al., 2003) to establish how sensitivity to color in human V1 depends on cortical depth. Stimuli with chromatic contrast and relatively low temporal frequencies will preferentially stimulate the P pathway (Derrington and Lennie, 1984; Hubel and Livingstone, 1990; Kaplan and Shapley, 1982), which projects from the dorsal layers of LGN into layer 4C*β* of V1 and then to the cytochrome oxidase-rich blobs in the deep and superficial layers 2/3 of V1. The M pathway, which is relatively color insensitive and tuned to higher temporal frequencies (Calkins et al., 1994; Chatterjee and Callaway, 2003; Martin et al., 1997), projects from the ventral layers of LGN into layer 4C*α* of V1, then to layer 4B, and then to the extrastriate cortex (Blasdel and Lund, 1983; Callaway and Wiser, 1996; Yabuta and Callaway, 1998; for a review on M, P, & K pathway connections, see Nassi and Callaway, 2009). Therefore, because red/green stimuli contrast-reversing at 0.5 Hz and the black/white stimuli alternating at 12 Hz will produce responses biased toward the P and M pathways, respectively, different depth-dependent fMRI responses are expected for the stimuli used in this study.

An additional goal of the present study was to measure how the relative contributions of responses driven by chromatic and achromatic stimuli change as a function of eccentricity in V1. Decreasing sensitivity to red-green color contrast with increasing eccentricity has been measured behaviorally (Anderson et al., 1991; Newton and Eskew, 2003; Mullen, 1991; Mullen and Kingdom, 2002), and it has been hypothesized that this eccentricity preference is due to decreased P inputs to peripheral V1 (Mullen and Kingdom, 1996; Vanni et al., 2006). Similarly, the transient channels of the M pathway are more sensitive to fast flickers, and behavioral studies show that human observers are more sensitive to fast flicker in peripheral vision (McKee and Taylor, 1984; Snowden and Hess, 1992). It has also been shown using 3T fMRI that fast flicker produces a consistent BOLD signal across eccentricity of V1, while slowly alternating stimuli elicit a stronger signal in the fovea and weaker signal in the periphery (Horiguchi et al., 2009). Thus, slowly alternating, chromatic stimuli should elicit the strongest fMRI responses in foveal regions of V1, with the relative response to rapidly alternating, achromatic stimuli increasing in peripheral V1.

The visual features that differentiate putative M and P pathways in V1 have also been shown to cause a repetitive alternating pattern of stripes along V2. Interleaved thin (color-selective), thick (stereo-selective), and pale (form-selective) stripes of oxidase staining have been found with a repeating pattern of pale-thick-pale-thin in V2 of NHP (An et al., 2012; Chen et al., 2008; Hubel and Livingstone, 1985; Livingstone and Hubel, 1987a; 1988; Lu and Roe, 2007, 2008; Salzmann et al., 2012; Tootell et al., 2004; Vanduffel et al., 2002; Xiao and Felleman, 2004). Similar stripe-based subdivisions have been found in human V2 for selectivity of several features like temporally-selective stripes (Dumoulin et al., 2017), disparity-selective stripes (Nasr and Tootell, 2016; Tootell and Nasr, 2017), and color-selective stripes (Nasr et al., 2016; Tootell and Nasr, 2017). These stripes terminate at the V1/V2 border. NHP studies have found at least three types of structures that project from V1 into V2: interblobs in the superficial layers of V1 project equally to thick and pale stripes; layer 4B of V1 has a significant projection to thick stripes; blobs in the superficial layers of V1 project to thin stripes (Burkhalter and Bernardo, 1989; Livingstone and Hubel, 1988; Nassi and Callaway, 2007; Roe and Ts’o, 1995; Sincich and Horton, 2002; Sincich et al., 2010; Tootell et al., 1983). Thus, in NHPs the M-pathway dominates thick stripes and the P pathway contributes more strongly to thin stripes.

NHP histology (Burkhalter and Bernardo, 1989; Nassi and Callaway, 2007; Sincich and Horton, 2002; Sincich et al., 2010; Tootell et al., 1983) and imaging studies (Li et al., 2019) have measured the spacing of these stripes to be about 4 mm from the center of a thick stripe to the center of a thick stripe. Human histology (Adams et al., 2007; Burkhalter and Bernardo, 1989; Hockfield et al., 1990; Tootell and Taylor, 1995) and neuroimaging studies (Dumoulin et al., 2017; Nasr and Tootell, 2016; Tootell and Nasr, 2017) have found these stripes to have spacing ranging from 4 mm to 8 mm from one thick stripe to its adjacent thick stripe. In addition to imaging the laminar profiles of responses to chromatic and achromatic stimuli as a function of eccentricity in V1, this study was able to verify the appearance of these stripes at the V1/V2 border.

## 2. Methods

### 2.1. Participants

Seven neurotypical adults (five females) aged 23 to 50 years old participated in the experiment. All experimental procedures were approved by the University of Minnesota’s Institutional Review Board. Written informed consent was obtained from all participants before the experiments, in accordance with the Declaration of Helsinki. Participants were compensated at a rate of $20 per hour. All participants were scanned twice in order to provide test/re-test validation. Two participants were scanned a third time due to high motion in one of the first two scans.

### 2.2. Apparatus

The stimuli were presented using a VPixx PROPixx projector. Participants wore polarized glasses that allowed for dichoptic presentation of the stimuli. The dichoptic presentation of the stimuli facilitated a separate, simultaneous experiment studying eye selectivity through the cortical depth. During any given block stimuli were presented to one eye (the background gray screen, with only a fixation mark, was presented to the other) at an effective frame rate of 60 Hz in the eye that was receiving stimulus.

The stimuli were projected onto a polarization-preserving screen placed in the magnet bore behind the participant’s head, which was viewed via a mirror situated above the participant’s eyes. The screen was 85 cm from the participant’s eyes, and the rectangle in which stimuli were presented was 46 cm × 26 cm.

### 2.3. Visual stimuli

**Fig. 1.**
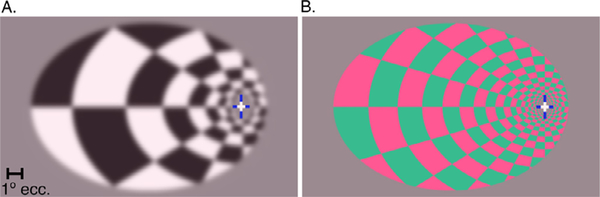
Examples of each stimulus display. A) Visual stimulus that targeted the M pathway (achromatic, lower spatial frequency, and higher temporal frequency (12 Hz). B) Visual stimuli that targeted the P pathway (lower luminance contrast, higher chromatic contrast, increased spatial frequency, alternating at 0.5 Hz).

Two types of visual stimuli were presented with the intent of differentiating responses in putative P and M pathways (Denison et al., 2014; Olman et al., 2012). Achromatic checkerboards with high contrast (70.3%) and lower spatial frequency (~1 cycle per degree (cpd) in the parafovea) flickering at higher temporal frequency (12 Hz) targeted the M pathway (Fig. 1A). The highest spatial frequencies were removed from the achromatic stimulus by blurring with a Gaussian kernel with a sigma of 0.2°. Chromatic (green and red) checkerboards with low luminance contrast (4.6%) and higher spatial frequency (check size was doubled, and edges were not removed by blurring) flickering at low temporal frequency (0.5 Hz) targeted the P pathway (Fig. 1B). The color values chosen for the chromatic stimuli were nominally isoluminant in CIELAB space and presented with a color calibrated system; actual luminance values were measured with a spectrophotometer through the polarized lenses worn by participants.

Typically, stimuli targeted at the M system have low contrast in order to provide weak drive to the P pathway. However, our previous work (Olman et al., 2012) found that the overall fMRI response amplitude to chromatic stimuli was much larger than the response to low-contrast achromatic stimuli (possibly because elaboration of the capillary bed is known to follow CO staining (Keller et al., 2011; Murphy et al., 2001), so we used higher contrast for the achromatic stimuli to elicit fMRI responses of similar magnitude in both conditions.

The checkerboard patterns were placed in an oval-shaped aperture on a gray background. A fixation cross was situated to one side of the stimulus; this design allowed stimulation out to 20° of visual angle on one side of the visual field (at the expense of the other). During the scan, participants were asked to maintain fixation on the cross and report via button press whenever the colors of the cross periodically reversed. The size of the checks was scaled so they were larger at higher eccentricities, doubling in size as eccentricity doubled from the fixation point into the periphery to roughly accommodate increasing receptive field sizes (and decreasing spatial frequency preferences) in both the putative M and P pathways.

It is known that the perceptual isoluminance point varies across the visual field (Bilodeau and Faubert, 1997; Livingstone and Hubel, 1987b; Mullen, 1985). The stimuli were not varied across the visual field to try to accommodate this variance because individual differences in cortical magnification, isoluminance, and subtle variations in the quality of the projected image tend to confound attempts to equate effective luminance contrast (unwanted drive to putative M pathways by the chromatic stimuli) as a function of eccentricity. Instead of measuring isoluminance values at several eccentricities for each observer and customizing the stimuli accordingly (Nasr et al., 2016), we chose to present stimuli that had low but non-zero luminance contrast throughout the visual field.

To confirm that the effective luminance contrast of the red/green colors used in the chromatic stimuli was between 5 and 10% across the range of eccentricities measured, we asked 3 of the observers to perform a contrast detection task in which either red and green patches or black and white patches were alternated in parafoveal, middle, or peripheral regions of the visual stimulus field at 30 Hz. The flicker detection threshold for the black/white colors was 7% of the level used for the main experiment (SD = 2%) in parafoveal regions, 4% (SD = 0.5%) at 5–15° eccentricity, and 5% (SD = 0.7%) beyond 15° eccentricity. These values equate to contrast detection thresholds of 3–5% luminance contrast.

For the red/green colors, the detection thresholds were 54% (SD = 3%), 48% (SD = 7%) and 50% (SD = 7%) of the levels used in the main experiment, in the parafoveal, middle, and peripheral regions, respectively. From this we conclude that the effective luminance contrast of the chromatic stimuli was twice the threshold, on average, and roughly 10 times lower than the measured 70% luminance contrast of the achromatic stimuli. In addition, we did not measure significant variation in the effective contrast across 1–20° eccentricity, consistent with the data in Bilodeau and Faubert (1997).

### 2.4. Data collection

Functional MRI data were collected at the University of Minnesota’s Center for Magnetic Resonance Research on a Siemens 7T scanner equipped with a custom-made head coil (32-channel transmit, 4-channel receive) (Adriany et al., 2012) that was used for T_2_*-weighted gradient echo (GE) echo-planar imaging (EPI). Images were acquired with a coronal orientation in 36 slices positioned near the occipital lobe. Image resolution was 0.8 mm isotropic (field of view (FOV): 129.6 mm × 160 mm; matrix size: 162 × 200); the data were acquired with an in-plane parallel imaging acceleration factor (R) of 3 and a right-left phase-encode direction (6/8 Partial Fourier, echo-spacing: 1.01 ms). The repetition time (TR) was 2 s and the echo-time (TE) was 23.4 ms.

Each functional scan consisted of four conditions: achromatic stimuli presented to the left eye (AL), achromatic stimuli presented to the right eye (AR), chromatic stimuli presented to the left eye (CL), and chromatic stimuli presented to the right eye (CR). Within one scan all conditions including rest blocks were presented four times in pseudorandom order. Each condition was presented in 16 s blocks so that the scan lasted 320 s. A total of 10 functional scans were conducted during a session.

During each scanning session, we also acquired (1) a short phase-encode reversed (left-right) EPI sequence to assist in distortion compensation during data preprocessing, and (2) a T_1_-weighted GE EPI (T_1_ wEPI) sequence scan (van der Zwaag et al., 2018). Since both the T_1_ wEPI data and the functional data were collected during the same session with the same resolution, sampling, and echo spacing, they were subject to the same distortion. The T_1_ wEPI sequence was used to define the gray matter (GM) in the functional data.

We acquired a whole-brain T1-weighted MP-RAGE (Mugler and Brookeman, 1990) during the session as an additional anatomical scan (1.0-mm isotropic, TR = 3100, TE = 3.27, flip angle = 6°, FOV = 156 × 192). A structural scan was acquired separately for all participants on a Siemens 3T scanner (0.8-mm isotropic T_1_-weighted MP-RAGE).

Each participant completed two additional population receptive field (pRF) mapping scans (Dumoulin and Wandell, 2008) for the purpose of retinotopic mapping in a separate scanning session. During the task, participants were asked to maintain fixation on a central point while a bar moved across the visual field at one of eight orientations (Left – Right, Top Left – Bottom Right, Top – Bottom, Top Right -Bottom Left, Right-Left, Bottom Right – Top Left, Bottom – Top, Bottom Left -Top Right) in forward and reverse directions (e.g., Top – Bottom vs. Bottom – Top), for a total of 16 directions. The moving bar was populated with dynamic and highly salient visual stimuli from one of three categories (faces, objects, or noise) flickering at either 2 or 12 Hz. The bar spanned the visual field (out to 8° eccentricity) and subtended 2° of visual angle in width. Each bar took 16 s to complete the movement across the visual field, and each bar sweep direction occurred once in a scan. There were 4 s of rest between each bar sweep and 4 s of rest at the beginning and end of each scan such that each scan took 324 s.

The two pRF scans used a GE EPI sequence that captured the whole brain. The pRF images were acquired at 1.4 mm isotropic resolution with a coronal slice orientation in 56 slices (FOV: 160 mm × 129 mm; matrix size: 114 × 92). The data were acquired with an in-plane parallel imaging acceleration factor (R) of 3 and a right-left phase-encode direction (6/8 Partial Fourier, echo-spacing: 1.01 ms). The repetition time (TR) was 2 s and the echo-time (TE) 22.6 ms.

### 2.5. Data pre-processing

A brief description of pre-processing steps is provided here; a fully detailed sample processing script is included with the data provided at https://openneuro.org/datasets/ds003043/

#### 2.5.1. 3T data

We segmented the 3T reference anatomy to define the GM/white matter (WM) boundary and the pial surface using FreeSurfer’s recon-all command (https://surfer.nmr.mgh.harvard.edu/, v6.0.0).

#### 2.5.2. Functional data

Functional data were processed using tools provided by AFNI (https://afni.nimh.nih.gov/afni, v18.2.04). Motion compensation was performed using AFNI’s 3dvolreg to register all scans to the mean image of the functional scan acquired before the distortion-compensation (reversed phase-encode) scan.

The 3T anatomy and the 7T T_1_ wEPI were aligned to the motion-compensated functional data. This was done by first registering the 3T anatomy to the 7T anatomy (coarsely aligned to the functional data) using 3dAllineate. This step generated a transformation matrix that was used to generate an initial registration of the 3T anatomical reference volume to the functional data, which was refined by a second call to 3dAllineate (lpc cost function). We processed the data from the 7T T_1_ wEPI by fitting each voxel’s intensity as a function of the slice-specific inversion time for each volume acquisition. The processed T_1_ wEPI (a T_1_ map) was then aligned to the functional data.

At this point, the cerebellum was stripped out of the T_1_ wEPI using the 3T anatomy as reference. We then segmented the T_1_ wEPI volumes using 3dSeg (initializing the segmentation with GM/WM/CSF masks derived from the FreeSurfer segmentation on the 3T anatomy) so that each voxel was classified as GM, WM, or cerebral spinal fluid (CSF). As the T_1_ wEPI was subject to the same distortion as the functional data but has better GM/WM contrast, this segmentation was used to define the GM in the functional data space.

Distortion compensation was performed for the functional data using the 3dQwarp command, using the T_1_ wEPI GM to generate a nonlinear WARP volume to optimize the GM registration between the functional and anatomical data. The WARP volume was then combined with the motion correction parameters to produce motion- and distortion-corrected fMRI data with a single resampling step.

A GM overlap mask was created by selecting the surface nodes where the T_1_ wEPI GM marker was present throughout at least 75% of the GM in the reference anatomy and projecting those nodes through the cortical depth (Weldon et al., 2019). This mask ensured that depth-dependent analyses were performed only in regions with good registration between functional and anatomical data, since depth information was derived from a separate anatomical scan.

A binary veins mask was created by taking the average signal-to-noise ratio (SNR) map processed functional data and marking any voxel with SNR below 11. Visual inspection verified that only voxels near large veins and on movement-prone edges of the brain were marked after this step (Olman et al., 2007). This mask was then projected through the cortical depth to mark voxels that should not be included in laminar analyses because they were underneath or adjacent to large veins.

#### 2.5.3. pRF data

The retinotopic mapping scans were pre-processed using a pipeline similar to the sub-millimeter data. Distortion compensation was executed using AFNI’s 3dQwarp function which nonlinearly warped the functional pRF scans with a phase-encode reversed reference scan. Motion compensation was performed using AFNI’s 3dvolreg function. The anatomical data were aligned to the corrected functional data with AFNI’s 3dAllineate function. The amplitude values of the functional data were converted to percent signal change and demeaned.

### 2.6. Data analysis

#### 2.6.1. pRF analysis and ROI delineation

pRF analyses were conducted using custom tools designed and implemented in AFNI (see, Silson et al., 2015). A detailed processing script is included with the data provided at https://openneuro.org/datasets/ds003043.

**Fig. 2.**
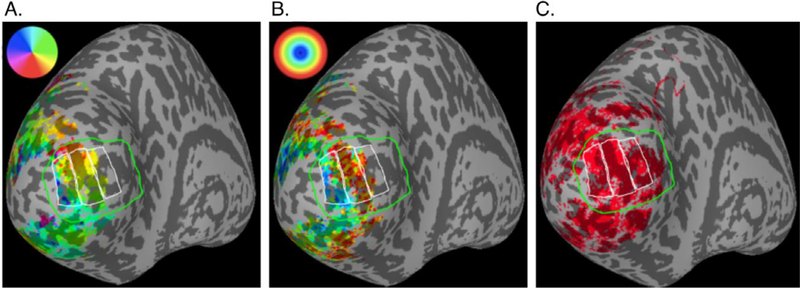
Retinotopic mapping of V1 and manually drawn ROIs. In each panel, the green line borders the probabilistic topography of V1 (Wang et al., 2015) and the white lines indicate the parafoveal (~2–4° eccentricity), middle (~4–8°), and peripheral (~8–20°) ROIs. A) The color overlay indicates polar angle estimated from pRF mapping scans. This was used to verify the location of the V1 border. B) The color overlay indicates estimated eccentricity from the pRF mapping scans, in which stimuli did not go beyond 8° eccentricity because the fixation point was in the center of the screen and the stimulus was circular. C) Red indicates a binary mask of activation from the main experiment (*p* < 0.001 single-voxel F-statistic, *p* < 0.001 after cluster-wise correction for multiple comparisons). In the functional scans, participants fixated on one side of the screen, so stimuli extended to 20° eccentricity. Therefore, the peripheral ROI was drawn past the extent of the retinotopy data to include the full extent of the data from the main experiment.

We used the resulting retinotopic maps to verify that the V1 boundaries defined by a publicly available probabilistic atlas were accurate (Wang et al., 2015). Using the V1 boundary and eccentricity maps generated from the pRF data as a guide, we manually segmented V1 into parafoveal, middle, or peripheral regions of interest (ROIs) (Fig. 2A).

The average size of the parafoveal ROIs was 1660 voxels (SD=207 *n* = 10); the average size of the mid-eccentricity ROIs was 1660 voxels (SD=282, *n* = 10); the average size of the peripheral ROIs was 1780 voxels (SD=289, *n* = 10). After restricting the ROIs to voxels associated with surface nodes where alignment was good, large veins were absent, activation was present throughout the cortical depth, and alignment was good, an average of 691 (SD=191, *n* = 10), 889 (SD=202, *n* = 10), and 732 (SD-216, *n* = 10) voxels were used for parafoveal, middle, and peripheral ROIs, respectively.

#### 2.6.2. General linear model analysis

The data were analyzed with a standard general linear model (GLM) using AFNI’s 3dDeconvolve to estimate the amplitude of response (percent signal change) during each of the four conditions (AL, AR, CL, CR) via linear regression against a model that was a hemodynamic response function [href = *t*^ 4* exp(-t)/(4^2* exp(−4)] convolved with a box-car function representing the 16-second blocks of stimulus presentation. Voxels not significantly modulated by visual stimulus presentation (*p* < 0.001, uncorrected; cluster-wise correction, *p* < 0.001) were excluded from further analyses. In addition, surface nodes for which significant modulation was not present in at least 75% of the GM depth under the node were excluded from depth-dependent analyses.

Although stimuli were presented separately to participants’ left and right eyes, to enable a separate study of ocular dominance, we collapsed across eye-of-presentation for the present analysis to estimate responses to chromatic and achromatic stimuli [*C* = (CL + CR)/2; *A* = (AL + AR)/2]. Selectivity for chromatic stimuli in each voxel was defined as the normalized difference in responses to chromatic and achromatic stimuli: selectivity = (C − A)/(*C* + *A*)/2.

#### 2.6.3. Depth-dependent analysis

For the depth dependent analyses, only data from the contralateral hemisphere (i.e. corresponding to the hemifield with more extensive stimulation) were analyzed. All voxels in an ROI were combined to provide a single estimate at each depth for each participant. We excluded any dataset with total motion greater than 2 mm (root-mean-square across the 3 Cartesian directions) and any dataset with fewer than 2500 significantly modulated voxels across the 3 ROIs. Four datasets were excluded due to excessive motion, and 2 were excluded due to an insufficient number of significantly modulated voxels; thus we included 10 out of the original 16 datasets in our subsequent analyses.

**Fig. 3.**
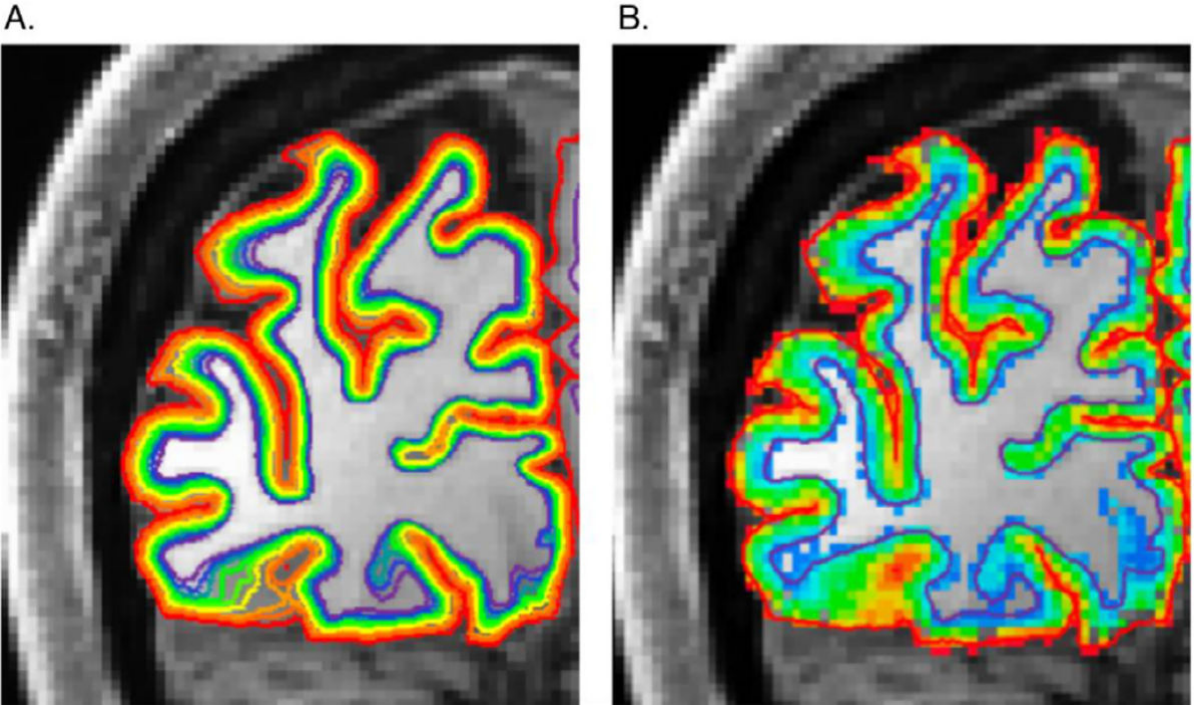
Assignment of gray matter and voxel depth locations. A) Each colored line represents a depth location inside the gray matter of one hemisphere relative to the white matter. B) All GM voxels are color-coded for their depth location where red is closest to the pial and purple is closest to the white matter.

We segmented the GM derived from the 3T anatomy into 10 depths using an equivolume solution (Waehnert et al., 2014) implemented in FreeSurfer (https://github.com/kwagstyl/surface_tools). These depths were projected into the space of the functional data (Fig. 3). Each functional voxel was assigned a depth depending on its registration to the anatomical GM. We used the fine segmentations with smoothed data only for visualizing the overall pattern of functional responses across GM and not for statistical analyses. At 0.8 mm isotropic, our resolution was too coarse to define depth bins that actually correspond to the 6 histological layers of the GM. Therefore, only the functional responses at the most superficial and deepest layers were used for depth-dependent statistical analyses.

There were five necessary components to create accurate depth-dependent profiles: hand-drawn ROIs (Fig. 2A), the GM overlap mask that ensured accurate functional/anatomical data registration for GM depth assignment, the significance mask, the vein-exclusion mask, and the GM depth assignment for each voxel. The GM overlap mask, significance mask, and vein-exclusion mask were combined to select the voxels analyzed within each ROI.

Statistical analysis was conducted with R Studio. Our dependent variable was selectivity for chromatic stimuli (i.e. percent signal change for the contrast (C − A)/(*C* + *A*)/2. A two-way analysis of variance was conducted to test for main effects of ROI (parafovea, middle, periphery) and Depth (Superficial or Deep layer) and for any interaction.

#### 2.6.4. Stimulus-selective band analysis

**Fig. 4.**
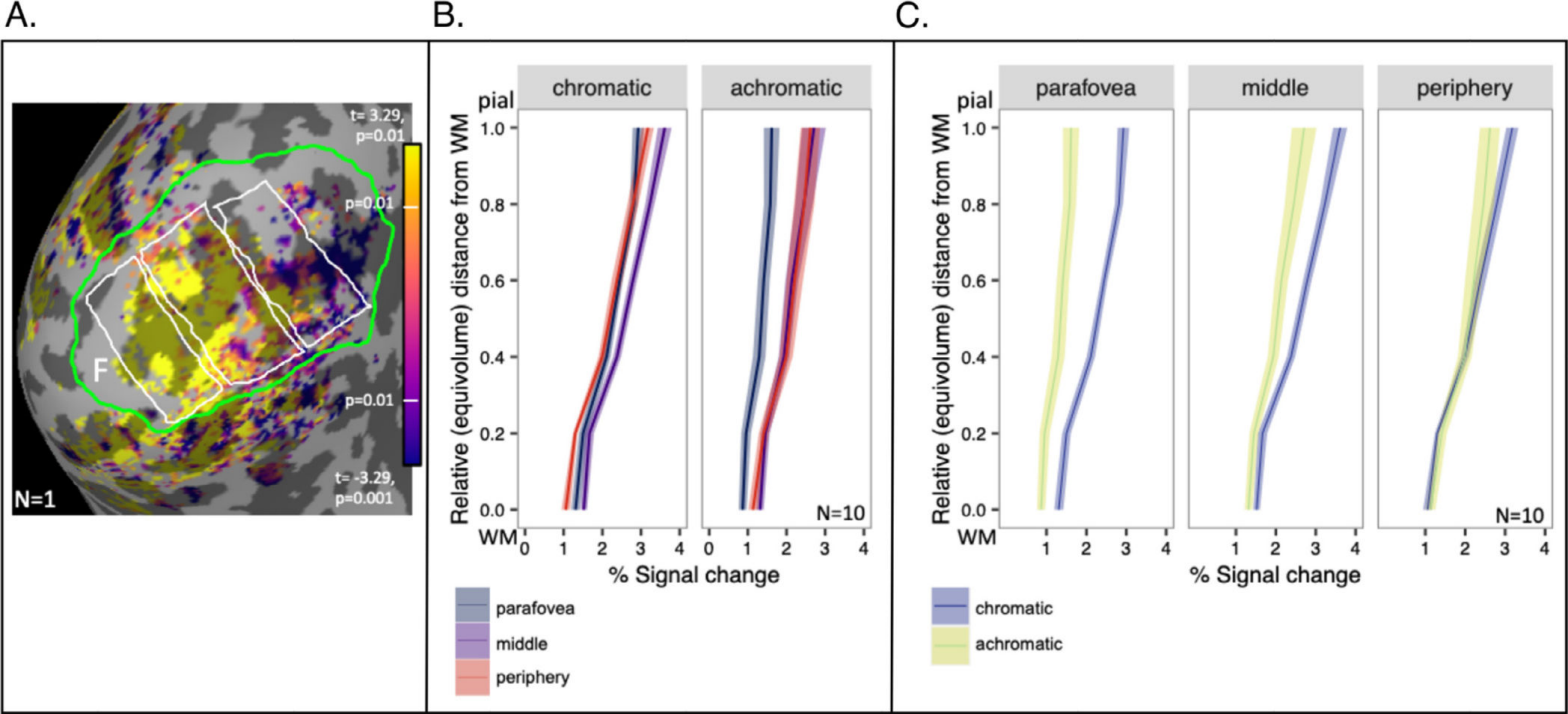
Responses to chromatic and achromatic stimuli in V1. A) T-statistics for chromatic selectivity are displayed for one participant (S1) on an inflated representation of the medial aspect of occipital cortex of the left hemisphere. Surface nodes with significant visual responses to all stimuli (*p* < 0.001, after cluster-wise correction) are displayed in color on an inflated representation of occipital lobe, where dark gray indicates sulci and light gray indicates gyri. Foveal retinotopic cortex is labeled with the letter “F”. Parafoveal, middle, and peripheral ROIs are indicated by white borders (parafoveal is the leftmost ROI adjacent to the fovea). The V1 boundary is indicated by a green border. B) Estimates of the magnitude of responses to chromatic and achromatic stimuli in each of the three ROIs. Data represent responses from the 10 datasets (hemispheres) meeting all inclusion criteria; shading indicates standard error of the mean. C) The same data as in (B) are plotted again, grouped so comparisons between chromatic and achromatic responses can be made within each of the 3 ROIs.

We observed alternating bands of activation selective to chromatic/achromatic stimuli along the dorsal and ventral border of V1 (Fig. 4). We characterized the size and pattern of these bands on each hemisphere for each scanning session by manually selecting the start and end of each band when visualizing the data using AFNI’s surface mapper (https://afni.nimh.nih.gov/Suma, v18.2.04). We manually marked the proximal and distal ends of each band (with proximal being defined as the end closest to the V1/V2 border) and quantified the distance between the center of bands for 6 datasets for which the bands were visible past the dorsal boundary of V1 border and for 8 quarterfield representations past the ventral boundary of V1.

## 3. Results

Both visual stimuli elicited robust responses throughout V1 (Fig. 4A), with the response to the achromatic stimulus increasing with increasing eccentricity (parafovea < periphery, *t* (19) = −4.433, *p*<0.001). On the other hand, responses to the chromatic stimulus showed no significant difference between the parafovea and periphery (*t* (19) = −0.002, *p* = 0.999) (Fig. 4B). The result of this was that the chromatic stimulus dominated parafoveal regions, and response differences were smallest in the peripheral ROI (Fig. 4C).

**Fig. 5.**
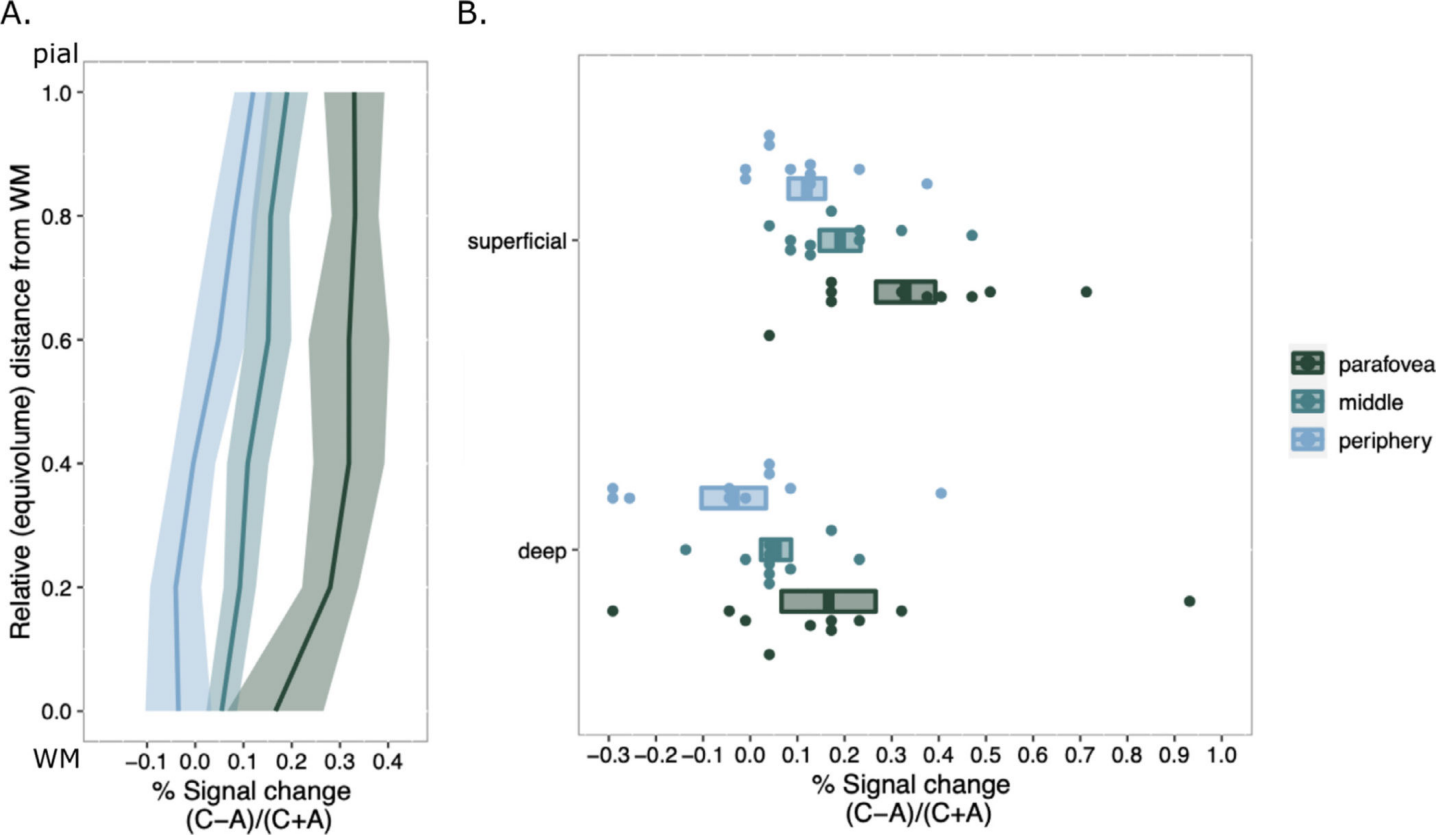
Laminar profiles of differential responses to chromatic vs achromatic stimuli in V1 as a function of eccentricity. (A) Differences were normalized by the average of both responses to eliminate dependence on overall BOLD response amplitude as a function of depth or eccentricity. Profiles were computed separately for parafoveal, middle, and peripheral ROIs. The shaded area around each profile is the standard error (*n* = 10). (B) Individual subject signal change is displayed as single points for the most superficial layer and the deepest layer. Significant differences are discussed in main text.

We characterized the difference between responses to chromatic stimuli and achromatic stimuli by computing a selectivity index, which was the difference between the two responses normalized by the average of the two responses. Even after normalization, stronger responses to chromatic stimuli were most pronounced in superficial layers (Fig. 5A). Because the fMRI voxels are relatively large compared to the GM thickness, statistical tests were only performed using the most superficial and deepest depth bins (Fig. 5B). There was a significant main effect of ROI (*F*(2, 18) = 10.413, *p* < 0.001, *η*p^2^ = 0.761) and a main effect of Depth (*F*(1, 9) = 29.159, *p* < 0.001, *η*p^2^ = 0.886) on chromatic selectivity with no interaction (*F*(2, 18) = 0.087, *p* = 0.917, *η*p^2^ = 0.225). This result demonstrates that chromatic selectivity varies across eccentricity in V1 and is significantly different in superficial and deep layers of the GM. A Bonferroni correction for multiple comparisons (*α* = 0.05/3 = 0.005) was applied to post hoc analyses following up the main effect of ROI (collapsed across Depth). Chromatic selectivity was greater in the parafoveal ROI than the peripheral ROI (*p* < 0.001), indicating chromatic selectivity decreased with eccentricity in V1. When analyzing chromatic selectivity across Depth (collapsed across ROI) we found greater chromatic selectivity in superficial layers than in deep layers (*p* < 0.001).

**Fig. 6.**
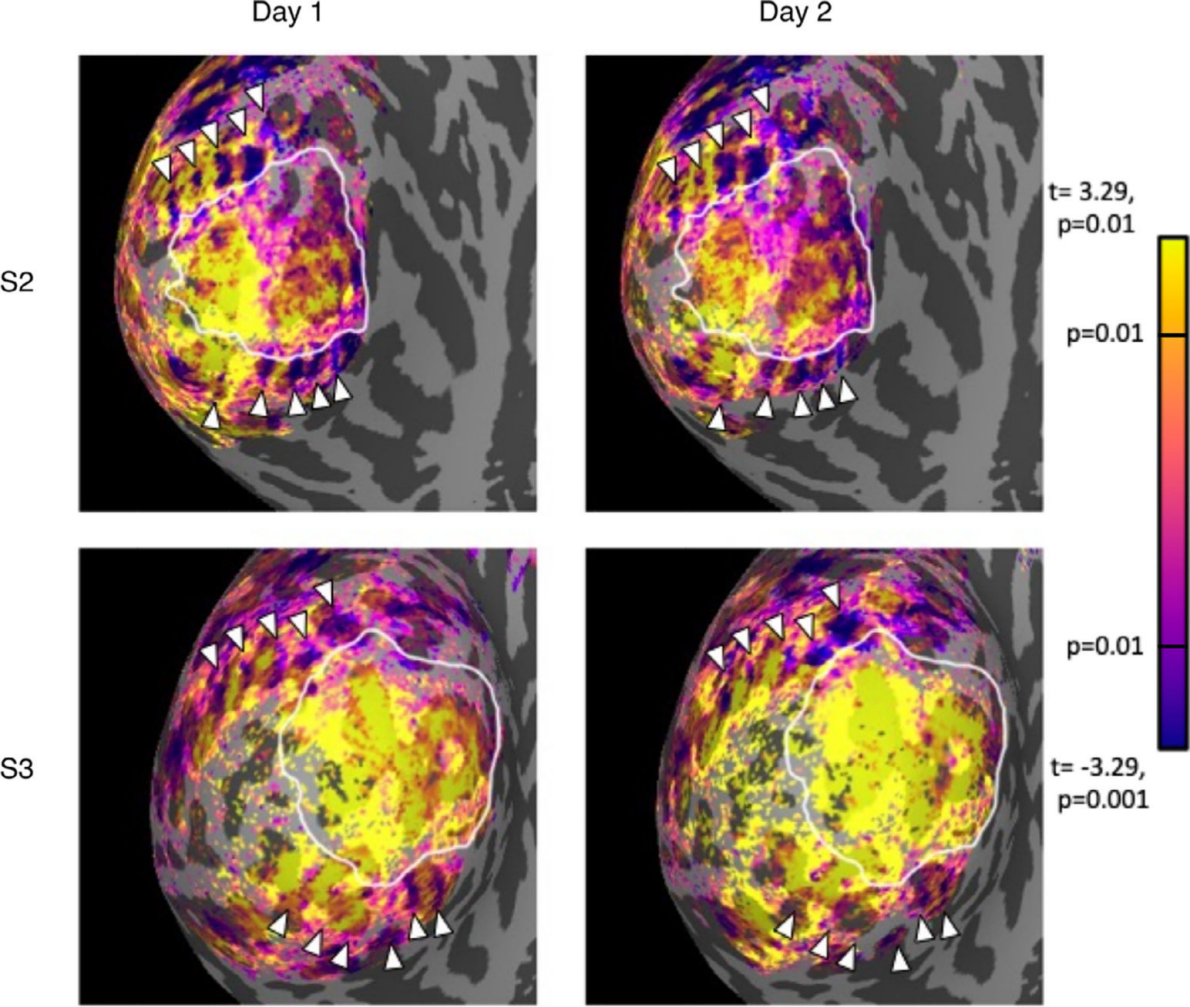
Repeatability of extrastriate bands across days for two participants. Each panel shows an inflated representation of the occipital lobe, where dark gray indicates sulci and light gray indicates gyri. The color overlay indicates the *t*-statistic associated with the chromatic-achromatic contrast is significant (*p* < 0.01, uncorrected). Yellow nodes represent significant chromatic selectivity and purple nodes represent significant achromatic selectivity. White arrows point to achromatic stimuli-selective bands that are consistent in location across days.

We observed periodic, stimulus-selective bands adjacent to and orthogonal to both the dorsal and ventral boundaries of primary visual cortex (Fig. 6), consistent with previous reports (Dumoulin et al., 2017; Nasr and Tootell, 2016; Tootell and Nasr, 2017). The average spacing of bands on the dorsal side of V1 was 7.5 mm (*SEM*=*0.32, n* = 6); the average spacing of bands on the ventral side of V1 was 7.8 mm (*SEM*=0.54, *n* = 8). These values are within the range of spacing (4–8 mm) reported from measurements in post-mortem human brains (Adams et al., 2007; Burkhalter and Bernardo, 1989; Hockfield et al., 1990; Tootell and Taylor, 1995). The bands were observed in the same locations in different scanning sessions for a given participant (Fig. 6), indicating that they are likely a true measure of the underlying neural architecture and not an imaging artifact. We also computed depth dependent analyses of chromatic selectivity within two band types (i.e., by treating bands selective for chromatic stimuli and bands selective for achromatic stimuli as different ROIs). We found no significant difference in laminar profiles of stimuli-selectivity between band types.

## 4. Discussion

In this work, we examined whether selectivity for slow-flickering, chromatic stimuli varied through the cortical depth. We found that responses to chromatic stimuli were larger than responses to achromatic stimuli in superficial GM, but not deep GM, even after normalizing by average percent signal change to account for superficial bias in T_2_* - weighted fMRI. This finding suggests that the underlying laminar profile of responses to chromatic stimuli is biased toward superficial layers, compared to the underlying laminar profile responses to high-contrast achromatic stimuli. This finding is consistent with a preliminary result previously found in humans (Olman et al. 2012).

One possible explanation for the apparent superficial bias for responses to the chromatic stimulus, relative to the achromatic stimulus, would be the location of cytochrome oxidase (CO) blobs to which color-sensitive neurons project. Those blobs are most evident in superficial layers 2 and 3 (Horton, 1984), and weakly present in deep layers 5 and 6 (Livingstone and Hubel, 1982). The superficial CO blobs sit higher in the GM than the Layer 4B neurons that are the primary V1 target of the M pathway neurons (in NHP) and are expected to respond more strongly to the achromatic stimuli than the chromatic stimuli.

The pial bias of the BOLD signal in superficial layers is a known challenge for depth-dependent analysis of GE data in particular (Uludağ and Blinder, 2018). To account for this, we took careful steps to minimize the possible influence of large surface vessels by removing voxels that had (vein-attributed) high SNR from analysis and normalizing activation differences at each depth by overall activation levels to verify that the P-selectivity in superficial layers was due to the stimuli and not an artifact due to the location of select voxels. Normalization, in particular, removes a significant portion of the superficial bias known to be present in the fMRI signal, but not all of it. The strongest evidence that the pial bias in chromatic selectivity is not merely an artifact of the imaging modality is that it varies with eccentricity throughout V1.

Another caveat for interpretation of the measured bias toward chromatic stimuli in superficial layers is that superficial signals can represent signals from middle and deep layers (Havlicek and Uludağ, 2020). It is possible that the bias is actually present in middle layers, but upward pooling of fMRI signal extends the bias to superficial layers. However, a superficial signal that is only inherited from deeper sources would not be larger than a signal in deeper sources, as we see in our data (Fig. 5), particularly in middle and peripheral ROIs. Thus, despite not including a depth-deconvolution step (Havlicek and Uludağ, 2020) by subtracting deep signals from middle signals in our analysis, we conclude that the positive bias in superficial layers in our data is a result of chromatic selectivity.

In addition to observing chromatic selectivity (preference for chromatic stimuli compared to achromatic stimuli) across depth, we also examined how chromatic selectivity varied with eccentricity. We found that parafoveal regions were more responsive to chromatic stimuli than achromatic stimuli, and peripheral responses to chromatic and achromatic stimuli were similar. Interestingly, the variation in chromatic selectivity was due to differences in achromatic responsivity: responsivity to red/green slow-flickering (0.5 Hz) stimuli was roughly constant across the visual field, and responsivity to achromatic, fast-flickering (12 Hz) stimuli increased with eccentricity. This result diverges somewhat from a demonstration of eccentricity dependence of temporal frequency sensitivity, where sustained flicker responsivity (roughly associated with the P pathway) declined with eccentricity, and transient flicker responsivity (roughly associated with the M pathway) was roughly constant across eccentricity (Horiguchi et al., 2009). However, our data are consistent with findings from a 3T fMRI study showing achromatic responsivity as relatively constant near the vertical meridians in V1 and increasing across the visual field along the horizontal meridian (Vanni et al., 2006). Our stimuli and ROIs avoided the vertical meridian, which maximized our sensitivity to this previously reported increase in responses to achromatic stimuli with increasing eccentricity. Responsivity to chromatic stimuli was roughly constant across eccentricity in our data, which is consistent with fMRI results showing red/green modulation to be evenly distributed up to 20° eccentricity when stimuli are corrected for cortical magnification (Vanni et al., 2006).

Our chromatic stimulus was not corrected for varying isoluminance values across eccentricity. The isoluminance point varies somewhat with eccentricity (Bilodeau and Faubert, 1997; Livingstone and Hubel, 1987b; Mullen, 1985); however, this variation (~5% increment or decrement in effective contrast over the central 20° of the visual field) is relatively small compared to the changes we measured with eccentricity. The variation in the isoluminance point across eccentricity likely contributed to the degree to which our chromatic stimuli evoked neural responses; however, we do not believe the spatial variation in isoluminance point across the visual field is enough to explain an increase responsivity to chromatic stimuli in regions beyond 10° eccentricity. A follow-up study controlling for the isoluminance variation would rule out this possible confound.

Our third finding was the expression of alternating, repeating bands of chromatic or achromatic selectivity stemming from the V1 border into V2 and V3. In multiple participants, we found chromatic and achromatic bands for both the dorsal and ventral sides of V1. In general, the bands in our data followed a pattern similar to bands described in past fMRI studies that concluded these alternating bands of activation were analogous to those found in functional imaging studies that used chromatic and achromatic stimuli (Nasr et al., 2016; Tootell and Nasr, 2017) or stimuli manipulating temporal frequency (Dumoulin et al., 2017). These bands were consistent in size and found across days for various participants. We characterized the size and pattern of these bands to understand if the patterns in our data entailed a similar kind of marker for color selectivity. The spacing between our bands was on the larger range previously reported averaging ~7.7 mm distance from the center to center of adjacent bands of the same type (e.g. thick-thick) compared to a 4–8 mm range distance reported in humans (Adams et al., 2007; Burkhalter and Bernardo, 1989; Dumoulin et al., 2017; Hockfield et al., 1990; Tootell and Taylor, 1995; Nasr and Tootell, 2016; Tootell and Nasr, 2017). This is expected, since the smaller end of that range comes from postmortem studies, and tissue contraction during histological preparation is expected. Furthermore, we found that the expression of these bands was stable across multiple days of scanning (Fig. 6).

The widths of the chromatic-selective and achromatic-selective bands in V2 of our study were roughly comparable. This finding is in line with studies reporting variable widths of thin/thick stripes in macaques (DeYoe et al., 1990; Hubel and Livingstone, 1987; Li et al., 2019; Roe and Ts’o 1997). The bands in our study are defined by the subtraction of two competing conditions, rather than isolated presentation of a single condition; therefore, the width of the bands will be determined by the relative strength of the stimuli in activating the different populations of neurons in the corresponding bands. Our stimuli were presented dichoptically, which would produce suboptimal responses in the stereo-selective thick V2 stripes (assuming human anatomy matches NHP anatomy) and reduce their apparent width. While the presence and color-selectivity of alternating bands suggests strong similarities between human V2 physiology and NHP physiology, our fMRI study, using differential methods cannot make specific claims about the relative widths of the bands in V2.

Our data join the ranks of about a dozen other datasets that test sub-millimeter fMRI against known underlying mesoscale neural architecture and conclude that fMRI, if used carefully, has the spatial specificity to distinguish the responses of neuronal responses separated by less than a millimeter. Previous work also showed, however, that if care is not taken in developing laminar profiles, errors are easily made in assigning depth to functional responses or interpreting depth-dependent profiles. Our analysis pipeline included steps that excluded regions where accurate alignment cannot be verified (Weldon et al., 2019) or where the presence of surface veins biased laminar profiles (Kashyap et al., 2018; Olman et al., 2010). The key to our ability to study the eccentricity dependence of M/P laminar profiles was using a T_1_-weighted EPI (van der Zwaag et al., 2018) to guide non-linear registration between functional and anatomical data across the entire area of the primary visual cortex. The functional data themselves do not have good enough contrast between GM and WM to guide non-rigid-body warping, and rigid-body warping can only optimize registration for a subset of the functional volume when significant distortions are present (distortion compensation from fieldmaps can be applied, but is never perfect). With this addition, however, we were able to generate laminar profiles across an extended region of interest with good confidence in their accuracy, because the segmented T_1_-weighted EPI also allowed computation of a metric of local registration quality (GM Overlap). This work has therefore demonstrated that sub-millimeter fMRI techniques are now robust enough to pursue large-scale depth profiling of cortical responses.
